# What Makes Individuals Stick to Their Exercise Regime? A One-Year Follow-Up Study Among Novice Exercisers in a Fitness Club Setting

**DOI:** 10.3389/fpsyg.2021.638928

**Published:** 2021-05-28

**Authors:** Christina Gjestvang, Frank Abrahamsen, Trine Stensrud, Lene A. H. Haakstad

**Affiliations:** ^1^Department of Sports Medicine, Norwegian School of Sport Sciences, Oslo, Norway; ^2^Department of Sport and Social Sciences, Norwegian School of Sport Sciences, Oslo, Norway

**Keywords:** fitness club members, motives, novice exercisers, physical activity, self-efficacy

## Abstract

**Objectives:**

A fitness club may be an important arena to promote regular exercise. However, authors have reported low attendance rates (10 to 37%) the first months after individuals sign up for membership. It is therefore important to understand the reasons for poor exercise adherence. In this project, we aimed to investigate different psychosocial factors that might increase the likelihood of reporting regular exercise the first year of a fitness club membership, including self-efficacy, motives, social support, life satisfaction, and customer satisfaction.

**Methods:**

New members (≤4 weeks membership, *n* = 250) classified as novice exercisers (exercise < 60 min/week the last 6 months) from 25 multipurpose gyms were followed for 1 year. Data were collected by an electronic survey including background and health factors, self-efficacy, social support, life satisfaction, motives, customer satisfaction, and exercise attendance, and was answered at start-up and after three (*n* = 224), six (*n* = 213), and 12 (*n* = 187) months. It is well established in the literature that ≥2 exercise sessions/week improve physical fitness in novice exercisers (if adhered to). Hence, we divided the participants into regular exercise attendance (≥2 sessions/week) and non-regular exercise attendance (≤1 session/week, exercise dropout, or membership dropout) in the analysis.

**Results:**

A mixed-effects logistic regression model revealed that the strongest predictor for reporting regular exercise attendance was higher levels of the motive “enjoyment” (OR = 1.84, *p* ≤ 0.001, 95% CI for OR = 1.35, 2.50), followed by self-efficacy “sticking to it” (OR = 1.73, *p* = 0.002, 95% CI for OR = 1.22, 2.46) and social support from friends and family (OR = 1.16, *p* ≤ 0.001, 95% CI for OR = 1.09, 1.23).

**Conclusion:**

In novice exercisers, regular exercise at three, six, and 12 months was associated with higher scores of the motive “enjoyment,” self-efficacy (“sticking to it”), and social support compared with non-regular exercise. Our results show that the majority of new fitness club members do not achieve regular exercise behavior.

## Introduction

Even though the health benefits of physical activity (PA) are well documented (Warburton and Bredin, 2017), research shows that 38% of the European adult population’s physical activity level is inadequate (Mayo et al., 2019). Thus, two public health challenges are to motivate inactive individuals to become physically active and to encourage already active individuals to increase or maintain their PA level. It is therefore important to examine factors that increase the probability of starting with and stay physically active and to identify factors that may reduce the risk of dropping out.

A fitness club holds equipment for group and individual exercise and represents one large context to be physically active (IHRSA, 2020). To date, this industry has about 185 million members and 210 000 gyms worldwide. Thus, fitness clubs are important arenas for the promotion of PA and exercise (IHRSA, 2020). We have previously reported that among new fitness club members, only 37% exercised regularly irrespective of activity setting, and only 17% used the gym twice weekly the first year as a member (Gjestvang et al.,2020a,b). Other authors have also reported low attendance rates (10 to 37%) the first three to 6 months after individuals sign up for gym membership (Middelkamp et al., 2016; Sperandei et al., 2016). Hence, it is important to understand the reasons for poor exercise adherence.

It is shown that individuals experience a wide range of psychosocial facilitators and barriers to regular exercise (Ayotte et al., 2010; Bauman et al., 2012; Choi et al., 2017; Scarapicchia et al., 2017). Most often reported correlates of exercise behavior are self-efficacy, social support, and different motives (such as “exercising for the inherent enjoyment” or “exercising for personal challenge”) (Ayotte et al., 2010; Bauman et al., 2012; Choi et al., 2017; Scarapicchia et al., 2017). Also, it is well established that satisfaction with life is positively associated with PA (Bize et al., 2007; Gillison et al., 2009). However, to our knowledge, no studies have investigated the association between these recognized psychosocial factors and exercise attendance among novice exercisers in a fitness club setting.

We have previously shown that regular exercisers had higher scores on motives such as “enjoyment” (e.g., “I enjoy the feeling of exerting myself”) and “challenge” (e.g., “To give me goals to work toward”), and life satisfaction compared with non-regular exercising members (Gjestvang et al., 2020a; Heiestad et al., 2020). Other authors have also suggested that higher levels of motives considered as intrinsic (Thogersen-Ntoumani and Ntoumanis, 2006; Kathrins and Turbow, 2010; Kopp et al., 2020), self-efficacy (Jekauc et al., 2015), and social support (Jekauc et al., 2015; Sas-Nowosielski and Szopa, 2015) contribute to regular use of the gym. A limitation of previous research is, however, the use of a piecewise approach, often including data of only one or two psychosocial factors in the analysis. Exercise is a complex behavior, as for this, several psychosocial factors need to be considered when investigating the reasons for adherence (Bauman et al., 2012). Also, one challenge in the interpretation of previous findings is that most studies were cross-sectional. To our knowledge, only two former studies in this field were prospective and there is a need for research with a longer time-frame than 20 and 30 weeks as in the previous studies (Jekauc et al., 2015; Kopp et al., 2020).

Members seek fitness clubs that will satisfy their specific needs, such as opening hours, equipment, and exercise concepts. Further, authors have shown that a satisfied member is more likely to attend the fitness club regularly (Ferrand et al., 2010; Gocłowska and Piątkowska, 2017). Hence, customer satisfaction is also a key factor to consider when understanding exercise attendance among fitness club members, especially in novice exercisers with limited gym experience and preferences.

Using data from the research project “Fitness clubs – a venue for public health?” (Gjestvang et al., 2017, 2019, 2020a; Haakstad et al., 2020; Heiestad et al., 2020), we aimed to investigate different psychosocial factors that might increase the likelihood of reporting regular exercise the first year of a fitness club membership, including self-efficacy, social support, motives, life satisfaction, and customer satisfaction. Our hypothesis was that self-efficacy, perceived motives considered as intrinsic, and social support would be higher in regular exercisers compared with those reporting non-regular exercise attendance.

## Materials and Methods

The research project Fitness clubs – a venue for public health?, was a 1-year prospective study conducted from October 2015 to October 2018 (Gjestvang et al., 2017, 2019, 2020a; Haakstad et al., 2020; Heiestad et al., 2020). The main aim was to increase evidence about the characteristics of those individuals who are able to stay active and continue with regular exercise in a fitness club setting. Measures of self-efficacy, social support, life satisfaction, and perceived motives for exercise were primary outcomes, whereas customer satisfaction was a secondary outcome. Except for data on perceived motives and life satisfaction, the data set used in this study are original for publication and have not yet been used yet.

The Nordic fitness club chain used to obtain data in this study consists of multipurpose gyms, including a wide range of exercise concepts, resistance and cardio-exercise rooms, group exercise classes, and personal training. The membership fees are from mid (55 USD) to high (120 USD), dependent on each membership profile, and members purchase a 12-month contract that cannot be canceled or a “pay as you go” contract. The fitness clubs have long reception opening hours (6 am to 10 pm), childcare, and focus on customer satisfaction. All new members from 25 gyms were invited to take part in the study by an email-invitation from the fitness club chain. Eligibility criteria were: ≥18 years, <4 weeks membership, untrained (exercising <60 min/week at moderate or vigorous-intensity in the last 6 months) (Garber et al., 2011), and healthy (no disease/illness considered to hinder exercise, e.g., severe heart disease or hypertension). A total of 676 new members wanted to participate in the study, of whom 148 did not respond after the first e-mail correspondence, and 278 did not meet the eligibility criteria (regularly exercising *n* = 270, disease/illness *n* = 8). Hence, 250 participants with equal gender distribution were included and followed for 1 year. More details of the research project are published elsewhere (Gjestvang et al., 2017, 2019, 2020a; Haakstad et al., 2020; Heiestad et al., 2020).

### Sample Size Calculations

Details of sample size calculations have been reported previously and found to be eligible (Gjestvang et al., 2020a; Heiestad et al., 2020). For the present study, with respect to a mixed effects logistic regression, including eight independent variables, a minimum of ten participants per predictor variable was assumed appropriate. Hence, we needed a minimum of 80 participants to conduct the analysis and aimed to recruit all new fitness club members (*n* = 250) who fulfilled the eligibility criteria between October 2015 to October 2017.

### Ethics Statement

The Norwegian Social Science Data Service provided approval for this study (NSD 44135). The project was reviewed by the Regional Committee for Medical and Health Research Ethics (REK 2015/1443 A) concluding that according to the Act on medical and health research (the Health Research Act 2008), the study did not require extensive review. All participants signed informed consent for participation following the Helsinki Declaration.

### Outcome Measures

A standardized electronic survey was answered by 250 at start-up, and 224, 213, and 187 after three, six, and 12 months follow-up, respectively. A total of 184 participants answered at all time-points. Losses to follow-up included life situation (*n* = 16), injury/disease (*n* = 6), relocation (*n* = 1) and unknown reasons (*n* = 43).

The questionnaire contained 52 questions at start-up and 65 questions at three, six, and 12 months. Additional questions at three, six, and 12 months covered exercise habits, use of the fitness club, and customer satisfaction. All questions were close-ended, and the survey took approximately 25 min to complete at each time-point. On all questions, the participants could tick “Does not apply” or “I do not want to answer,” which was treated as missing data in the analysis. For the present study, the participants answered questions concerning background and health factors (such as age, gender, total household income, occupation, and education), and psychosocial factors (self-efficacy, social support, life satisfaction, and perceived motives) at start-up. At three, six, and 12 months follow-up, the participants reported on the same psychosocial factors, as well as customer satisfaction and exercise attendance. We asked the participants to answer the questions over the last 4 weeks, due to potential recall bias associated self-report (Vetter and Mascha, 2017). The questionnaire sections used to answer the present study aims are shown in Table 1.

**TABLE 1:** Psychosocial factors and customer satisfaction.

	Specifics	Questions/statements	Response options†	Scores
Background*		Age, gender, body weight, level of education, total household income, cohabitation, and occupation.	–	–
Self-efficacy**	Twelve statements on how confident an individual was in a range of conditions. Statements were divided into two subscales, and a sum score for each subscale was calculated.	**Sticking to it:** *“Stick to your exercise program after a long, tiring day at work,” “Exercise even though you are feeling depressed,” “Continue to exercise with others even though they seem too fast or too slow for you,” “Stick to your exercise program when undergoing a stressful life change (e.g., divorce, death in the family, moving),” “Stick to your exercise program when your family is demanding more time from you,” “Stick to your exercise program when you have household chores to attend to,” “Stick to your exercise program even when you have excessive demands at work,” “Stick to your exercise program when social obligations are very time consuming.”* **Making time for exercise:** *“Get up early, even on weekends, to exercise,” “Set aside time for a physical activity program; that is walking, jogging. swimming, biking, or other continuous activities for at least 30 min, three times per week,” “Attend a party only after exercising,” “Read or study less in order to exercise more.”*	*“1 I know I cannot,” “2,” “3 Maybe I can,” “4,” “5 I know I can.”*	1 to 5. Higher scores indicate greater self-efficacy for exercise.
Social support**	Six statements regarding how often, over the previous four weeks, an individual’s family/friends had been supportive of them exercising. All statements were amassed, and total score was calculated.	*“Exercised with me,” “Gave me encouragement. to stick with my exercise program,” “Complained about the time I spend exercising.” “Planned for exercise on recreational outings.” “Helped plan activities around my exercise,” “Asked me for ideas on how they can get more exercise.”*	*“1 None,” “2 Rarely,” “3 A few times,” “4 Often,” “5 Very often.”*	6 to 30. Higher scores demonstrate greater social support for exercise.
Motives**§	Thirty five statements on how a motive was a personal motive for exercise. Statements were allocated into 14 subscales, and a sum score for each subscale was calculated.	**Enjoyment:** *“Because I feel at my best when exercising,” “For enjoyment of the experience of exercising,” “Because I find exercising satisfying in and of itself,” “Because I enjoy the feeling of exerting myself,”* **Challenge:** *“To give me goals to work toward,” “To give me personal challenges to face,” “To develop personal skills,”* **Revitalization:** *“Because it makes me feel good,” “To recharge my batteries.”* **Stress management:** *“To give me space to think,” “Because it helps to reduce tension,” “To help manage stress.”* **Affiliation:** *“To make new friends,” “To spend time with friends and to enjoy the social aspects of exercising,” “To have fun being active with other people.”*	*“0 Not at all true for me,” “1,” “2,” “3,” “4,” “5 Very true for me.”*	0 to 5. Greater score indicates the importance of a motive.
Life satisfaction**§	Five statements related to satisfaction with the individuals’ life as a whole. All statements were amassed, and a total score was calculated.	*“In most ways my life is close to my ideal,” “The conditions of my life are excellent,” “I am satisfied with life,” “So far I have gotten the important things I want in life,” “If I could live my life over, I would change almost nothing.”*	*“1 Strongly Disagree,” “2 Disagree,” “3 Slightly Disagree,” “4 Neither Agree nor Disagree,” “5 Slightly Agree,” “6 Agree,” “7 Strongly Agree.”*	5 to 35. Higher score demonstrates higher life satisfaction.
Customer satisfaction***	Fifteen statements on how satisfied the individual was with the fitness club. Statements were assembled into four areas of the club’s functioning, and a sum score for each was calculated.	*“How satisfied are you with the following conditions at your fitness center?”:* **Service:** *“Introduction and guidance,” “Opening hours,” “price of membership fee,” “Service quality,” “The atmosphere“*. **Facilities:** *“Square meters“*, *“Wardrobes,” “Parking conditions,” “Maintenance and cleaning,” “Quality of equipment.”* **Group exercise classes/instructors:** *“Group exercise instructors,” “Quality of group exercise classes,” “Group exercise class schedule.”* **Personal trainers:** *“Personal trainers,” “Quality of personal trainers.”*	*“1 Very dissatisfied,” “2 Dissatisfied,” “3 Neutral,” “4 Satisfied,” “5 Very satisfied.”*	1 to 5. Higher scores demonstrate greater service satisfaction.
Exercise attendance***		*“Have you been exercising regularly?”*, *“How often have you exercised per week on average at the fitness club?*.”	*“Yes”* or *“No*,” and *“number of sessions.”*	–

*Answered at start-up of fitness club membership, **Answered at all time-points, ***Answered after three, six and 12 months follow-up, §Total score of life satisfaction and five motivational subscales significantly higher in regular exercisers (≥2 exercise sessions/week) compared with non-regular exerciser in our previous research were included in the present study.

Table 1 summarize questions and response options used to answer the present study aim. Assessment of self-efficacy was based on a validated version of the Self-Efficacy Survey (Sallis et al., 1988). The questionnaire assesses how confident an individual is to increase or continue with regular exercise in a wide range of conditions. The original scale consists of two subscales, with a total of 12 statements (Sallis et al., 1988). Each subscale covers four to eight statements where the participants rated each statement on a five-point scale. For each subscale, a sum score (from 1 to 5) was calculated by adding scores from each statement, divided by the number of statements.

Social support for exercise was based on a modified validated version of a social support questionnaire developed by Sallis et al. (1987), consisting of 13 statements concerning social support. The individuals rate each statement on how often, on a five-point scale, their family or friends have been supportive of them exercising. Due to seven statements considered with similar wording (such as “Asked me for ideas on how they can get more exercise” and “Discussed exercise with me”), six out of the total 13 statements were used in the present study. Since the questionnaire as a whole was comprehensive, the two sections family and friends were also merged. The scoring on the six statements was assembled, and a total social support score was calculated (from 6 to 30), where higher scores demonstrated greater social support for exercise.

The questionnaire section regarding life satisfaction was based on the validated Satisfaction of Life Scale (SWLS) (Diener et al., 1985), a short survey assessing satisfaction with the individuals’ life as a whole. SWLS contains five statements that the individual rates on a seven-point scale and a total score is calculated by adding scores from each statement (from 5 to 35), where higher scores demonstrated higher life satisfaction.

Perceived motives for exercise were based on the validated Exercise Motivations Inventory-2 (EMI-2) (Markland and Ingledew, 1997), assessing a broad range of exercise motives. The original EMI-2 comprises 14 subscales, with a total of 51 statements (Markland and Ingledew, 1997). Each subscale contains one to four statements where the individuals rate the significance of each statement as a personal motive for exercise on a six-point scale. A sum score (from 0 to 5) for each subscale is calculated by adding scores from each statement, divided by the number of statements. We have previously reported that total score of life satisfaction and five motivational subscales were significantly higher in regular exercisers compared with non-regular exercisers. Hence, in this study, total score of life satisfaction and the five motivational subscales were included (Table 1).

Data on customer satisfaction was based on a former questionnaire used in a Danish fitness club setting (Pedersen et al., 2011 in Danish), containing 15 statements. We categorized the statements into four subscales, including two to five statements in each subscale. The participants rated how satisfied they were with the fitness club’s functioning, on a five-point scale. By adding the score from each statement divided by the number of statements, a sum-score (from 1 to 5) for each subscale was calculated.

The questionnaire sections concerning Exercise Self-efficacy Scale, social support for exercise, SWLS, and EMI-2 were translated into Norwegian by three members of the research group, using a forward-backward translation technique. A bilingual Australian Associate Professor with English as mother tongue assured the final questionnaire sections by comparing the “new” English version with the original version. Based on this, some adjustments were made.

To obtain data on exercise attendance the participants reported on exercise frequency at the fitness club (Table 1). In line with definitions from Hawley-Hague (Hawley-Hague et al., 2016) and due to that ≥2 exercise sessions/week may improve physical fitness in novice exercisers (Garber et al., 2011), in the analysis, we divided the participants at each time-point into regular exercise attendance: reporting ≥2 exercise sessions/week and non-regular exercise attendance: reporting ≤1 exercise session/week, exercise dropout, or membership dropout.

### Statistical Analysis

Data were analyzed using SPSS (IBM Corp. Released 2016. IBM SPSS Statistics for Windows, Version 24.0. Armonk, NY: IBM Corp) and STATA Statistical Software (StataCorp. Released 2019. Stata Statistical Software, Version 16.0. TX: StataCorp LP.). An independent *t*-test for continuous variables, chi-squared test for proportions, or a repeated measures ANOVA were used as appropriate. We also calculated Cohen’s D effect size to determine what potential group differences practically mean (Cohen, 1988).

Pearson’s correlation coefficient was calculated for the association between the psychosocial factors, customer satisfaction, and exercise attendance. At each follow-up, Pearson’s correlation coefficients revealed correlations between regular exercise attendance and six psychosocial factors. Hence, we decided to use a mixed effects logistic regression with exercise attendance as a binary response variable (1 = regular exercise attendance, 0 = non-regular exercise attendance), to estimate the odds of regular exercise attendance associated with the six psychosocial factors as independent variables (Jaeger, 2008). Independent variables tested in the full model were; self-efficacy (“sticking to it” and “making time for exercise”), social support, and three motivational subscales (“revitalization,” “enjoyment,” and “challenge”). Based on significant differences between regular and non-regular exercisers, the model was adjusted for two background factors (gender and BMI classification). The model included a random intercept to account for unmeasured individual differences in the probability of exercise attendance. Few (*n* = 31) were categorized as regular exercisers throughout all the follow-ups, hence, this sample size was not large enough for the regression analysis. The mixed effects logistic regression therefore contained data from three, six, and 12 months, including 228 participants with 2.6 observations (time-points) on average.

Results are presented as means ± SD, or frequencies (n) and percentages, correlations coefficient (r), odds ratio (OR), 95% CI for OR, and effect sizes (d). Effect sizes were interpreted as small (0.20), medium (0.50), and large (0.80) (Cohen, 1988). A two-tailed alpha level of 0.05 was used for statistical significance and was adjusted as appropriate for the mixed effects logistic regression (*p* = ≤ 0.01).

## Results

Most of the participants (78.4%) was of Norwegian descent, with a mean age of 36.4 ± 11.3 years. The distribution of regular exercise attendance (≥2 sessions/week at the gym) at three, six, and 12 months were as follows: 51.8, 37.6, and 37.4%. About 17% reported regular exercise attendance at all time-points. At 12 months follow-up, 86.6% were still fitness club members. More data on background and health factors, physical fitness, PA level, and exercise attendance are described elsewhere (Gjestvang et al., 2017, 2019, 2020a; Haakstad et al., 2020; Heiestad et al., 2020).

At each follow-up, an independent *t*-test showed that the self-efficacy subscales “sticking to it” (mean diff: 0.60 to 0.74, *d* = 0.28 to 0.71) and “making time for it” (mean diff: 0.41 to 0.54, *d* = 0.32 to 0.55), social support (mean diff: 2.15 to 2.54, *d* = 0.17 to 0.54), and three motivational subscales [(“revitalization” (mean diff: 0.45 to 0.69, *d* = 0.38 to 0.59), “enjoyment” (mean diff: 0.85 to 0.91, *d* = 0.48 to 0.70), and “challenge” (mean diff: 0.74 to 0.79, *d* = 0.45 to 0.56)] were rated higher among those classified with regular exercise attendance compared with those attending non-regularly (*p* = ≤ 0.01). Pearson’s correlation coefficients also revealed that these six psychosocial factors were positively associated with regular exercise attendance, with correlations (r) ranging from 0.17 to 0.38 (Table 2).

**TABLE 2:** Pearson’s correlations between regular exercise attendance and psychosocial factors and service satisfaction.

	Regular exercise attendance		
	Three months (*n* = 224)	Six months (*n* = 213)	12 months (*n* = 187)
**Self-efficacy**			
Sticking to it	0.38**	0.29**	0.30**
Making time for exercise	0.24**	0.25**	0.18*
**Social Support**	0.24**	0.27**	0.25**
**Motives**			
Enjoyment	0.31**	0.32**	0.30**
Challenge	0.24**	0.23**	0.26**
Revitalization	0.18**	0.17*	0.27**
Stress Management	0.18**	0.21**	0.10
Affiliation	0.12	0.25**	0.13
**Life Satisfaction**	0.04	–0.001	0.21**
**Service Satisfaction**			
Service	–0.04	0.11	0.01
Facilities	0.02	–0.04	–0.02
Group exercise classes/instructors	0.09	0.12	–0.02
Personal trainers	0.01	–0.10	0.08

*Correlations significant at the 0.05 level, **Correlations significant at the 0.01 level. Regular exercise attendance =≥2 exercise sessions/week.

When putting all significant psychosocial factors into one model, adjusting for gender and BMI classification, a mixed-effects logistic regression showed that participants with a higher score in the motive “enjoyment,” self-efficacy (“sticking to it”), and social support were more likely to report regular exercise attendance (Table 3). The strongest predictor of reporting regular exercise attendance was higher levels of the motive “enjoyment” (OR = 1.84), followed by self-efficacy “sticking to it” (OR = 1.73) and social support (OR = 1.16).

**TABLE 3:** Mixed Effects Logistic Regression and Odds Ratio (OR) for reporting regular exercise attendance (*n* = 228).

Factor	OR	*p*	95% CI* for OR	
			Lower	Upper
Gender (female)	0.92	0.772	0.52	1.63
BMI classification	0.97	0.450	0.90	1.05
**Self-efficacy**				
Sticking to it	1.73	0.002	1.22	2.46
Making time for exercise	1.09	0.563	0.81	1.47
**Social support**	1.16	<0.001	1.09	1.23
**Motives**				
Enjoyment	1.84	<0.001	1.35	2.50
Challenge	1.04	0.716	0.83	1.30
Revitalization	0.76	0.079	0.56	1.03
**Constant**	0.01	<0.001	0.00	0.13

*Confidence interval. Regular exercise attendance =≥2 exercise sessions/week.

All participants were generally pleased with the member service, and no differences were found between regular and non-regular exercise attendance at the different time-points (Table 4). “Group exercise classes/instructors” (3.7 to 4.0) and “personal trainers” (3.5 to 3.9) were rated highest at each time-point. There was a drop in satisfaction score for “service” (3.6 and 3.4, mean diff: 0.21, *p* ≤ 0.001) and “group exercise classes/instructors” (4.0 and 3.7, mean diff: 0.26, *p* = 0.045) from 3 to 12 months follow-up.

**TABLE 4:** Customer satisfaction at the fitness club.

Variable (1 to 5)				
**Three months**	**Regular exercise (*n* = 116)**	**Non-regular exercise (*n* = 108)**		

	**mean ± SD**	**mean ± SD**	***p***	**Cohen’s d**

Service	3.59 ± 0.76	3.65 ± 0.64	0.574	0.09
Facilities	3.42 ± 0.67	3.39 ± 0.75	0.793	0.04
Group exercise classes/instructors	4.02 ± 0.78	3.88 ± 0.75	0.276	0.18
Personal trainers	3.91 ± 1.14	3.89 ± 1.03	0.905	0.02

**Six months**	**Regular exercise (*n* = 80)**	**Non-regular exercise (*n* = 133)**		

Service	3.58 ± 0.80	3.40 ± 0.78	0.104	0.23
Facilities	3.31 ± 0.80	3.38 ± 0.75	0.547	0.09
Group exercise classes/instructors	4.04 ± 0.85	3.84 ± 0.75	0.175	0.25
Personal trainers	3.50 ± 1.28	3.76 ± 1.11	0.286	0.22

**12 months**	**Regular exercise (*n* = 70)**	**Non-regular exercise (*n* = 117)**		

Service	3.41 ± 0.83	3.39 ± 0.73	0.891	0.03
Facilities	3.24 ± 0.69	3.28 ± 0.79	0.721	0.05
Group exercise classes/instructors	3.24 ± 0.69	3.28 ± 0.79	0.721	0.05
Personal trainers	3.65 ± 1.32	3.43 ± 1.16	0.461	0.18

*Regular exercise attendance =≥2 exercise sessions/week.*

## Discussion

The main finding in our study was that higher levels of the motive “enjoyment,” self-efficacy (“sticking to it”), and social support were the strongest predictors associated with reporting regular exercise attendance throughout the first year of a fitness club membership. We found no association between customer satisfaction and regular exercise attendance. The findings in this study were in line with our hypothesis, that self-efficacy, perceived motives considered as intrinsic, and social support would be higher in those reporting regular exercise attendance than those exercising irregularly. This indicates that among novice exercisers in a fitness club setting, higher levels of self-efficacy, intrinsic motives, and social support have the potential to positively influence exercise attendance.

Our results mirror studies of general PA among both children and adults in that motivation, self-efficacy, and social support are three of the strongest factors associated with PA behavior (Ayotte et al., 2010; Greaves et al., 2011; Bauman et al., 2012; Choi et al., 2017; Rhodes et al., 2017b; Scarapicchia et al., 2017). Comparable results are also found in the scarce literature concerning the fitness club industry, with one cross-sectional study (Kathrins and Turbow, 2010) and two prospective studies (Jekauc et al., 2015; Kopp et al., 2020) reporting that gym members with higher levels of self-efficacy, motives considered as intrinsic, as well as social support, were more likely to exercise regularly. Despite different study design and questionnaires than in our study, this suggests that members with intrinsic motivation may have a more autonomous foundation contributing to sustained exercise compared with members with more controlled motivation (such as extrinsic reasons for exercise) (Teixeira et al., 2012; Rodrigues et al., 2018). Kopp et al. (2020) found that controlled motivation was unrelated to use of the fitness club, whereas intrinsic motivation predicted self-reported attendance at the gym (Kopp et al., 2020). That said, there is no conclusive evidence that implicates the direction of causality in our findings. For instance, we cannot determine whether participants reporting regular exercise attendance were exercising because they had higher levels of self-efficacy, or whether they scored higher on self-efficacy since they exercised and perceived a feeling of mastery (Jekauc et al., 2015; Mikkelsen et al., 2017).

The way members perceive encouragement by significant others may create a strong normative support, and past experience with exercise might influence self-efficacy for exercise (Rhodes et al., 2017a). It is also proposed that social support positively influence exercise attendance by improving self-efficacy for exercise (Ayotte et al., 2010). Hence, initiating supervised group activities and social support in a safe setting with qualified instructors, may aid compliance to exercise among fitness club members (Hancox et al., 2018; Rodrigues et al., 2018). One study found higher exercise adherence in participants conducting a 12 weeks resistance exercise program with supervision from a personal trainer, compared with those exercising individually (Rustaden et al., 2017). A personal trainer may support the member in setting up easily achievable goals that may help improve self-efficacy and to focus on making exercise “enjoyable” instead of focusing on burning calories or weight loss (Rhodes et al., 2017a; Rodrigues et al., 2018). Yet, to date, the evidence is scarce regarding a personal trainer’s influence on an individual’s exercise behavior. We also believe that most novice exercisers may have a low level of knowledge on how to perform endurance and resistance exercise and implementation of exercise habits in their everyday life. Thus, it may be important to guide members in exercise planning and how to self-monitor progress toward personal goals, preferably with a cognitive-behavioral approach (Rhodes et al., 2017a). For instance, Annesi (2003) investigated new fitness club members receiving a 36-week cognitive-behavioral change treatment (guidance in goal setting, relapse prevention, and self-reinforcement) or a typical exercise counseling (guidance around types and dose of exercise) (Annesi, 2003). Their findings showed that the treatment group had higher exercise attendance (55% versus 36%) and less dropout (20% versus 55%) compared to the control group (Annesi, 2003).

Even though regular exercisers scored higher on the motive “enjoyment,” self-efficacy (“sticking to it”), and social support compared with non-regular exercisers, the distribution of regular exercise attendance decreased throughout the follow-up from 52 to 37%. Further, only 17% reported exercise at all time-points, an interesting finding considering that 86.6% were still gym members after 1 year. Hence, they paid a monthly membership fee to the club without using its facilities. One explanation for the high number of membership retention may be that most participants in the current study reported purchasing a 12-month contract that could not be canceled. It can be questioned whether a financial commitment and access to exercise equipment contribute to regular exercise. Based on the many positive health benefits of exercise (Rhodes et al., 2017a), the low prevalence of regular exercise attendance among fitness club members is worrying (Middelkamp et al., 2016; Sperandei et al., 2016; Gjestvang et al.,2020a,b). One explanation for the decrease in regular exercise attendance in our study may be seasonal variation, especially in participants recruited during fall/winter. Other authors have shown among the general US adult population that weekly PA level was greater during spring and summer than winter and fall (Pivarnik et al., 2003). However, the contrary may happen in Scandinavia. Due to low outdoor temperature, a member may have a medium to high exercise attendance at the gym during winter and fall, with a decreasing attendance during spring and summer because of more participation in outdoor activities. We have previously shown that when we combined exercise attendance both at the fitness club and at other arenas, the attendance rate was still decreasing throughout the follow-up (Gjestvang et al., 2020a). Hence, another explanation may be that some authors have reported that in individuals who experience a decrease in social support by family/friends or self-efficacy, this may lead to decreased exercise attendance (Martikainen et al., 2002; Bauman et al., 2012). Numerous research among young adults has also shown that a decrease in social support from significant others and self-efficacy to cope with barriers may be one explanation for a decline in PA level (Keating et al., 2005). As shown in our study, we believe that even minor changes in perceived motivation, self-efficacy, and social support may affect exercise attendance. We suggest that this should be emphasized in the fitness club industry, to counteract the poor exercise adherence among the 184 million individuals exercising in fitness clubs worldwide (IHRSA, 2020).

We did not find any association between customer satisfaction and regular exercise attendance at the fitness club, and this contrasts studies showing that regular use of the gym reflects the members’ satisfaction with the services offered (Ferrand et al., 2010; Gocłowska and Piątkowska, 2017). The fitness club chain we used to recruit participants focuses largely on customer satisfaction to provide strong customer relationships. The gyms offer several exercise concepts, a wide range of exercise equipment, group exercise classes, personal training, and in addition, long reception opening hours and childcare. Thus, these factors are possibly satisfying the member’s specific needs. Hence, we were not surprised that most participants reported medium to high customer satisfaction, which could explain the low membership dropout at 12 months follow-up (13.4%).

### Strengths and Limitations

A sample size of 250 participants, equally men and women, an electronic questionnaire primarily based on former validated surveys (Diener et al., 1985; Sallis et al., 1988; Sallis et al., 1987; Markland and Ingledew, 1997), several follow-ups during the first year of fitness club membership, as well as a high response rate at all follow-ups (*n* = 184, 73.6%) may be considered strong aspects of our study. Even though long-term regular exercise attendance might have to be verified in more than 1 year, our 1-year design made it possible to collect data in a longer time frame than previous research. Study limitations are that we gathered exercise attendance by self-report, with no objective data (such as membership card swipes) and that we should have included members from different fitness club segments. Hence, the generalizability of our findings to other gyms such as low-cost and Crossfit gyms are therefore uncertain. For instance, it may be differences in background factors (such as age, household income, and occupation), motivation, and self-efficacy between those joining a multipurpose fitness club and those joining a low-cost gym. Also, a multipurpose gym focus to a large extent on customer satisfaction compared with a low-cost gym. Even though we used a forward-backward translation technique for the questionnaire sections concerning self-efficacy, social support, life satisfaction, and motives, another limitation is that these instruments were not psychometrically tested and evaluated for the Norwegian language or a fitness club setting. Further, very few (*n* = 31, 17%) reported regular exercise attendance at all follow-ups, hence, our statistical power to conduct prospective data analyses was limited. Lastly, our quantitative design with numeric results may be too narrow to explain the complex aspect of exercise behavior.

## Conclusion

Among novice fitness club members, those exercising regularly at three, six, and 12 months had higher scores on the motive “enjoyment” and self-efficacy (“sticking to it”). Also, social support from family and friends was greater in those reporting regular exercise. Our results show that most new fitness club members use the gym intermittently and do not achieve a regular exercise behavior. Hence, there is a need for research investigating possible effective interventions in a fitness club setting, contributing to that members find interest and time to incorporate exercise in their everyday lives, as such prevent abandonment of exercise.

## Data Availability Statement

The raw data supporting the conclusions of this article will be made available by the authors, without undue reservation.

## Ethics Statement

The studies involving human participants were reviewed and approved by the Norwegian Social Science Data Services (NSD 44135). The project was reviewed by the Regional Committee for Medical and Health Research Ethics (REK 2015/1443 A) concluding that according to the Act on medical and health research (the Health Research Act 2008), the study did not require extensive review. The patients/participants provided their written informed consent to participate in this study.

## Author Contributions

LH conceived the idea for the research project, supervised the project, and wrote the protocol together with CG, FA, and TS. CG was responsible for participant follow-up, data collection and analysis, and outlined the manuscript. LH, FA, and TS contributed to interpretation of data, and revised the manuscript critically for important intellectual content, including English editing. All authors read and corrected draft versions of the manuscript and approved the final version.

## Conflict of Interest

The authors declare that the research was conducted in the absence of any commercial or financial relationships that could be construed as a potential conflict of interest.
